# Uric acid–driven NLRP3 inflammasome activation triggers lens epithelial cell senescence and cataract formation

**DOI:** 10.1038/s41420-024-01900-z

**Published:** 2024-03-09

**Authors:** Hong Liang Lin, Sheng Wang, Kota Sato, Yu Qiao Zhang, Bei Ting He, Jing Xu, Toru Nakazawa, Yong Jie Qin, Hong Yang Zhang

**Affiliations:** 1 Department of Ophthalmology, Nanfang Hospital, Southern Medical University, Guangzhou, China; 2 Department of Ophthalmology, Tohoku University Graduate School of Medicine, Sendai, Japan; 3 Department of Ophthalmology, Guangdong Provincial People’s Hospital (Guangdong Academy of Medical Sciences), Southern Medical University, Guangzhou, China; 4 School of Medicine, South China University of Technology, Guangzhou, China; 5 Department of Ophthalmic Imaging and Information Analytics, Tohoku University Graduate School of Medicine, Sendai, Japan; 6 Department of Ophthalmology & Visual Sciences, The Chinese University of Hong Kong, Hong Kong, China; 7 Department of Retinal Disease Control, Tohoku University Graduate School of Medicine, Sendai, Japan; 8 Department of Advanced Ophthalmic Medicine, Tohoku University Graduate School of Medicine, Sendai, Japan; 9 Department of Collaborative Program for Ophthalmic Drug Discovery, Tohoku University Graduate School of Medicine, Sendai, Japan

**Keywords:** Lens diseases, Senescence, Experimental models of disease, Inflammasome

## Abstract

Excessive uric acid (UA) is associated with age-related cataract. A previous study showed that a high UA level in the aqueous humor stimulated the senescence of lens epithelial cells (LECs), leading to cataract progression. To better understand the underlying mechanisms, we investigated UA-driven senescence in human lens tissue samples obtained during surgery, rat lens organ cultures, and in vivo experiments, using senescence-associated β-galactosidase (SA-β-gal) staining, electronic microscopy, Western blotting, and histological analyses. Initially, we identified markedly higher expressions of NLRP3 and caspase-1 in the lens capsules of hyper-uricemic patients compared to normo-uricemic patients. This increase was accompanied by a significant rise in the SA-β-gal positive rate. We next built a cataract model in which rat lenses in an organ culture system were treated with an increasing dosage of UA. Notably, opacification was apparent in the lenses treated with 800 μM of UA starting on the fifth day. Mechanistically, UA treatment not only significantly induced the expression of NLRP3, caspase-1, and IL-1β, but also upregulated the levels of SA-β-gal and the senescence regulators p53 and p21. These effects were fully reversed, and lens opacification was ameliorated by the addition of MCC950, a selective NLRP3 antagonist. Moreover, an in vivo model showed that intravitreal UA injection rapidly induced cataract phenotypes within 21 days, an effect significantly mitigated by co-injection with MCC950. Together, our findings suggest that targeting the UA-induced NLRP3 inflammasome with MCC950 could be a promising strategy for preventing cataract formation associated with inflammageing.

## Introduction

Lancet Global Health in 2020 reported that cataract remained the largest cause of blindness worldwide, responsible for approximately 45% of cases (over 15 million cases in 2020) [1]. Although cataract prevalence has declined due to increased surgical interventions [2], avoidable blindness caused by cataract in people older than 50 years nevertheless increased from 2010 to 2019 [1], likely due to aging populations. Given the large global health burden and limited resources, identifying risk factors is crucial for developing novel strategies to prevent cataract progression. As a multifactorial oculopathy, cataract is affected by individual, metabolic, and environmental factors [3]. Age, sex, race, and genes are important but non-modifiable factors for cataract formation [3]. Thus, special attention should be paid to modifiable risks, like diet and systemic diseases [4]. For instance, cataract is more common in individuals with high-glycemic diets or those exposed to ultraviolet-B radiation [5–7]. Diabetes patients have higher cataract morbidity even at a younger age [8]. Renal impairment and metabolic disorders also strongly accelerate cataract formation [9, 10]. However, although clinical cohort research has been conducted, microenvironmental changes in the aqueous humor related to cataract have rarely been studied. Previously, we reported that uric acid levels in the aqueous humor and serum were strongly correlated with cataract incidence [11]; however, the precise underlying mechanism remains unknown.

Uric acid (UA), as the end product of purine degeneration, was formerly considered to be an antioxidant in hydrophilic environments [12]. Hyper-uricemia is associated with gout, cardiovasculopathy, and kidney disorders; elevated UA can trigger inflammatory or immune reactions [13–15]. The pro-inflammatory nature of UA is related to the crystallization of monosodium urate (MSU); this has been clearly demonstrated in gout [13], which is a form of inflammatory arthritis characterized by increased white blood cells in the synovial fluid, resulting from the deposition of MSU crystals within joints [13]. The exact process by which MSU crystals induce inflammation was unclear until 2006, when Fabio Martinon et al. identified the role of the NLRP3 (nucleotide-binding oligomerization domain–like receptor pyrin domain containing–3) inflammasome [16]. Upon stimulation with elevated UA, NLRP3 oligomerizes and binds to ASC (apoptosis-associated speck-like protein containing a caspase recruitment domain), an adapter protein, and effector pro-caspase-1, ultimately forming the NLRP3 inflammasome [17]. In this cytoplasmic, multiprotein complex, pro-caspase-1 is cleaved into caspase-1, which then activates cytokines IL-1β and IL-18 in macrophages [17]. Consequently, persistent NLRP3 dysregulation leads to chronic inflammation, potentially resulting in pathological changes. The lens, as an avascular organ, is susceptible to changes in its metabolism and the transparency of the adjacent aqueous humor [18]. Any alteration in the composition of the aqueous humor can trigger or accelerate cataract development. Our previous findings indicated a strong association (*r* = 0.9) between hyper-uricemia and excessive UA in the aqueous humor, as well as urate deposition in the lens cortex [11]. We speculated that elevated UA levels contribute to the progressive clouding of the originally clear lens by activating the NLRP3 inflammasome.

The current study aimed to further elucidate the mechanisms behind UA-induced senescence in lens epithelial cells (LECs) and the subsequent formation of cataract. We observed that the NLRP3 inflammasome was enhanced in the LECs of patients with hyper-uricemia. This observation was corroborated by experiments based on a rat lens culture system and an in vivo model.

## Results

### Subject characteristics

The study enrolled a total of 14 eyes of 14 individuals diagnosed with age-related cataract. Of these, seven patients had hyper-uricemia, while the others exhibited normal serum UA levels (i.e., normo-uricemia). As summarized in Table 1, the hyper-uricemic subjects had a mean serum UA level of 521.67 ± 78.70 μmol/L, significantly higher than the 248.63 ± 39.93 μmol/L in the normo-uricemic subjects (*p* < 0.0001). The average age of the hyper-uricemic patients was slightly younger than the normo-uricemic individuals (62.43 ± 5.50 vs. 68.71 ± 4.64 years, *p* = 0.0401), even though they had cataracts with similar severity. As expected, aqueous humor UA levels were higher in the hyper-uricemic group (176.21 ± 24.26 μmol/L) compared to the normo-uricemic group (92.47 ± 10.37 μmol/L, *p* < 0.0001). No statistical differences were observed between the two groups in terms of gender, serum HbA1c level, or cataract grade (*p* > 0.1, Table 1).

**Table 1:** Characteristics of cataract patients with normo-uricemia or hyper-uricemia.

Parameters	Normo-uricemia (*n* = 7)	Hyper-uricemia (*n* = 7)	*p* value
Gender (n, M/F)	4/3	4/3	1.0000
Age (years)	68.71 ± 4.64	62.43 ± 5.50	0.0401*
sUA (μmol/L)	248.63 ± 39.93	521.67 ± 78.70	<0.0001*
ahUA (μmol/L)	92.47 ± 10.37	176.21 ± 24.26	<0.0001*
HbA1c (%)	5.30 ± 0.24	5.49 ± 0.32	0.2402
Scr (μmol/L)	57.50 ± 12.88	88.23 ± 8.21	0.0002*
Cataract grades	2.14 ± 0.38	2.43 ± 0.45	0.2225

Data were presented as mean ± SD with *Student’s t*-test or Mann–Whitney *U-*test.

*n* number, *sUA* serum uric acid, *ahUA* aqueous humor uric acid, *Scr* serum creatinine, / not applicable.

*p < 0.05, statistical difference between normo-uricemia and hyper-uricemia.

### NLRP3 inflammasome activation in cataract patients with hyper-uricemia

**Fig. 1:**
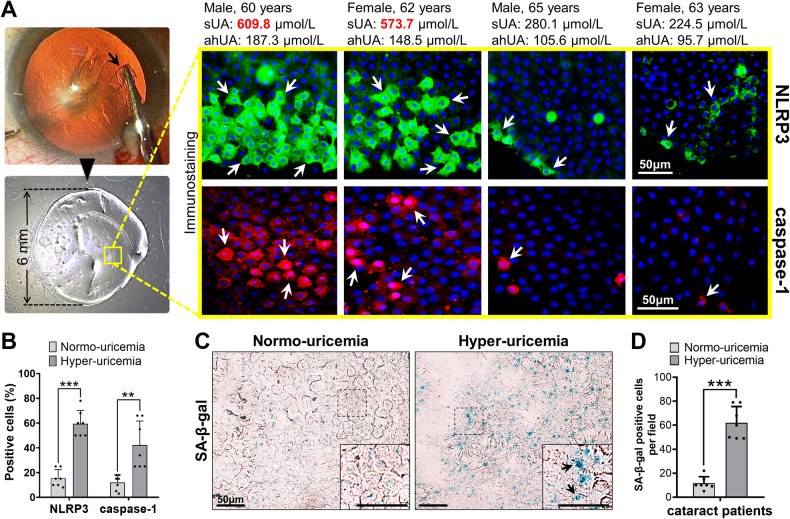
Hyper-uricemic individuals exhibited upregulation of the NLRP3-caspase-1 axis and aggravated cellular senescence. **A** Anterior lens capsules (shown by the black arrows) were harvested during cataract extraction and then flat-mounted for NLRP3 staining (with green fluorescence) and caspase-1 staining (with red fluorescence). The positive immunoreactivity is shown by the white arrows (scale bars = 50 μm). sUA serum uric acid, ahUA aqueous humor uric acid. **B** The number of NLRP3- and caspase-1-positive cells was quantified by calculating the ratio versus cells stained with DAPI. **C** Immunohistochemistry showed that SA-β-gal labeling (shown by the black arrows) was more distinct in the samples from the hyper-uricemic patients than those from the normo-uricemic patients (scale bars = 50 μm). **D** Quantitative analysis of the SA-β-gal-positive cells in each field was also performed. Mean ± SD, *n* = 7. *p < 0.05, **p < 0.01, ***p < 0.001.

To assess whether NLRP3 inflammation was upregulated in hyper-uricemic patients, we determined levels of related molecules in anterior lens capsules obtained from cataract surgeries (Fig. 1A, shown by the black arrow). Samples from hyper-uricemic patients exhibited stronger immunoreactivity in NLRP3 labeling, indicating an enhanced inflammasome response. Caspase-1, which functions downstream of NLRP3, was clearly detected in the hyper-uricemic patients but not in those with normo-uricemia (Fig. 1A, shown by the white arrows). The percentage of NLRP3- and caspase-1-positive cells was 60.86 ± 13.80% and 42.29 ± 19.37%, respectively, in the hyper-uricemic patients, which was much higher than in the patients with normo-uricemia (15.43 ± 7.04% and 12.00 ± 6.11%, respectively; *p* < 0.0001 for NLRP3, *p* = 0.0019 for caspase-1, Fig. 1B). Moreover, senescence staining with SA-β-gal showed higher positivity in samples with higher aqueous humor levels of UA than in samples with normo-uricemia (62.00 ± 13.54% vs. 18.14 ± 9.17%, respectively, *p* < 0.0001, Fig. 1C, D). Notably, these findings suggest that excessive UA in the aqueous humor may encourage NLRP3 inflammasome formation and LEC senescence.

### Modeling UA-induced cataract with organ culturing of rat lenses

**Fig. 2:**
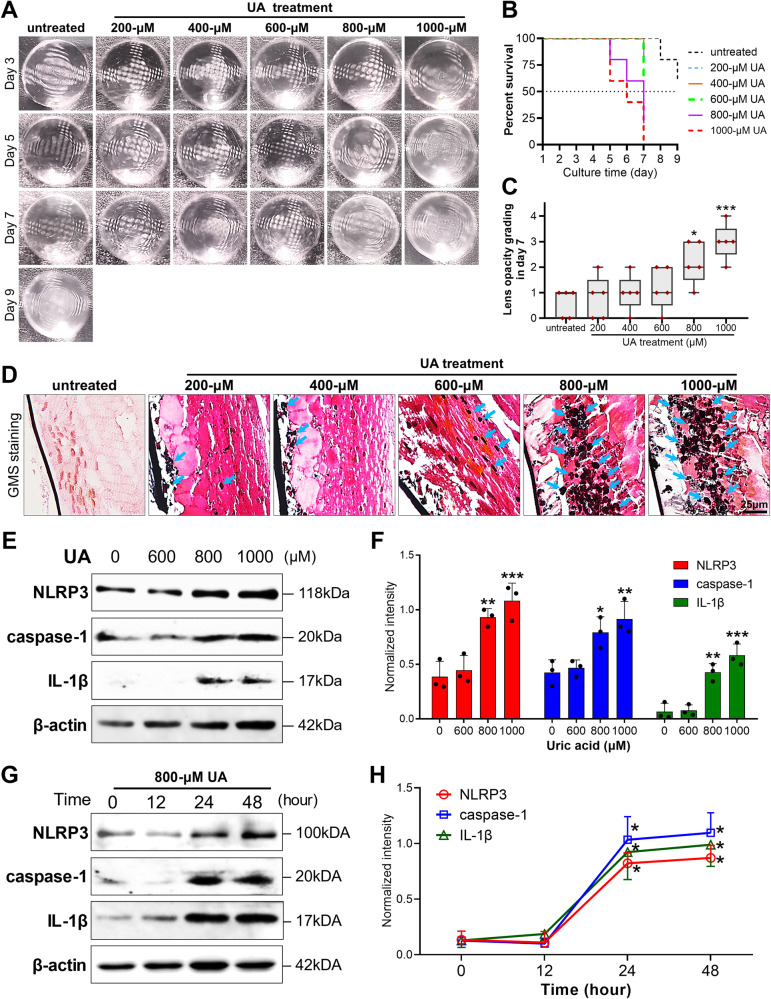
Excessive UA treatment facilitated lens opacification and activation of NLRP3 signaling during organ culturing of rat lenses. **A** Isolated rat lenses were treated with a gradient of increasing doses of UA (200 to 1000 μM) for 7 days; this duration was chosen because even untreated lenses stably preserved their transparency for only one week. **B** Definitive opacification (grade ≥2) occurred mostly in the lenses treated with 800 and 1000 μM UA, but it also occurred, although rarely, in the 200, 400, and 600 μM UA groups. **C** Lens opacity grades on day 7 rose remarkably when the UA dose reached 800 μM. Mean ± SD, *n* = 5. **D** GMS staining revealed urate deposition in the cortex lentis on day 7. Urate-labeled puncta (shown by the blue arrows) were noticeable in the lenses treated with 800 or 1000 μM UA (scale bars = 25 μm). UA uric acid, GMS Gomori methenamine silver. **E**, **F** Immunoblot analysis of the NLRP3/caspase-1/IL-1β pathway in lenses treated with a gradient dosage of UA (at 600, 800, and 1000 μM) for 7 days and corresponding quantification of the data. **G**, **H** Another immunoblot analysis was performed to investigate the temporal effect of 800 μM UA treatment on the expression of NLRP3, cleaved caspase-1, and mature IL-1β. Bands were analyzed with densitometry. Mean ± SD, *n* = 3. *p < 0.05, **p < 0.01, ***p < 0.001 compared with untreated controls.

To investigate the impact of aqueous humor UA concentration on cataract incidence, we developed an ex vivo rat lens experiment. Rat lenses were isolated for organ culturing and treated with a series of UA dosages (200 to 1000 μM) to simulate UA-induced cataract. Considering that rat lens transparency lasted for at most 1 week, even without any treatment (Fig. 2A, B, survival rate <60% on day 9), the observation was set at 7 days. As shown in Fig. 2A, by day 3, all cultured lenses were clear or slightly hazy (grade ≤1), developing varying degrees of opacification (grade ≥2) by day 5. Survival analysis demonstrated that when the UA dosage reached 800 μM or higher, clear lenses were almost all lost on day 7 (Fig. 2B). In contrast, in the groups that were treated with UA ranging from 200 to 600 μM, ≥50% of the translucent lenses survived (Fig. 2B). Likewise, a specific analysis on day 7 revealed no significant change in lens opacity grade for the 200 to 600 μM UA groups, while the 800 to 1000 μM UA groups exhibited distinctly higher grades than the controls (both *p* < 0.01, Fig. 2C). Additionally, GMS staining showed that lenses treated with 800 or 1000 μM UA had more evident urate-labeled puncta in the equatorial epithelia (indicated by the blue arrows in Fig. 2D), a feature rarely detected in other groups.

### UA activates NLRP3/caspase-1/IL-1β signaling in cultured rat lenses

To further clarify the impact of 800 μM UA on NLRP3 signaling, we collected and analyzed capsular epithelial samples from cultured lenses treated with gradient doses of UA (600, 800, and 1000 μM UA). Immunoblotting revealed that treatment with 800 or 1000 μM UA dramatically increased the levels of active NLRP3, cleaved caspase-1, and mature IL-1β compared to untreated controls (*p* = 0.0005 for NLRP3, *p* = 0.004 for caspase-1, and *p* = 0.0001 for IL-1β), but 600 μM UA in the rat lenses did not significantly induce expression of the above proteins (all *p* > 0.05, Fig. 2E, F). Additionally, further immunoblot analysis outlined the temporal profile of inflammasome activation: it potentially precedes lens clouding, as remarkable increases in NLRP3, caspase-1, and IL-1β were observed 24 and 48 h after 800 μM UA treatment (all *p* < 0.05, Fig. 2G, H). This indicates that 800 μM UA could continuously activate NLRP3/caspase-1/IL-1β signaling, leading to an inflammatory response during cataract development.

### Aggravation of LEC senescence in rat lenses cultured with excessive UA

**Fig. 3:**
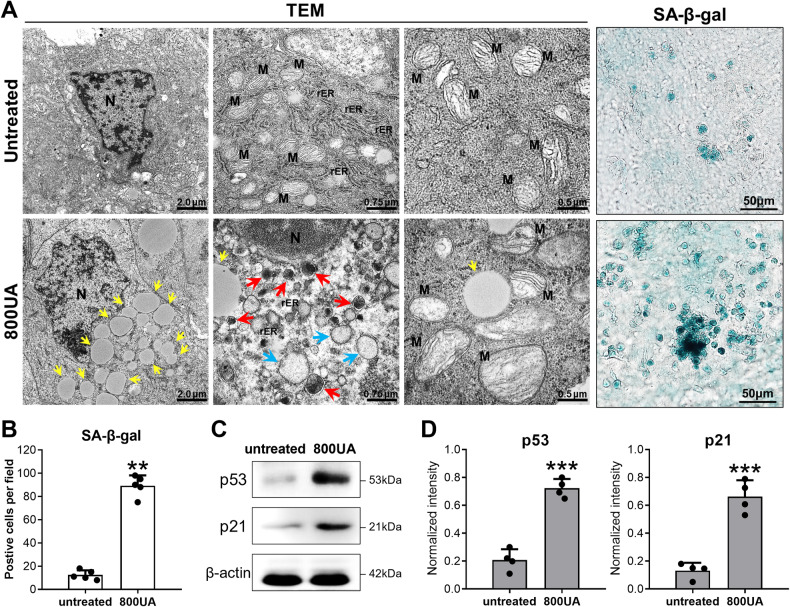
Addition of 800 μM UA facilitated rat lens senescence after 7 days of organ culturing. **A** TEM analysis and SA-β-gal staining of epithelial cells from the lenses treated with 800 μM UA, demonstrating augmentation of cellular senescence and organelle dysfunction (scale bars are shown in the figure). N nucleus, M mitochondrion, rER rough endoplasmic reticulum, LD lipid droplet, 800UA 800 μM uric acid treatment, TEM transmission electron microscopy. **B** Quantitative analysis of SA-β-gal-positive cells in lenses treated with or without 800 μM UA. Mean ± SD, *n* = 5. **C**, **D** Immunoblot analysis of the expression of senescence markers was performed after 7 days of incubation. It showed significant expression of p53 and p21 in rat lenses that received 800 μM UA. Mean ± SD, *n* = 3. *p < 0.05, **p < 0.01, ***p < 0.001 compared with untreated controls.

Tunneling electron microscope observations showed notable changes in cultured rat lenses after treatment with 800 μM UA, manifesting as increased aberrant lysosomes (shown by the red arrows in Fig. 3A), accumulated lipids (shown by the yellow arrows in Fig. 3A), decreased endoplasmic reticulum, vacuolization (shown by the blue arrows in Fig. 3A), and swelling of dysfunctional mitochondria. This morphology aligned with senescent-cell features, also evidenced by the rising number of SA-β-gal-positive cells per field in the 800UA group compared to untreated controls (89.20 ± 8.87 vs. 12.40 ± 4.04 cells, *p* < 0.0001, Fig. 3A, B). Moreover, the senescent biomarkers p53 and p21 were markedly upregulated after treatment with 800 μM UA, in contrast to their expression in untreated controls, which was low (both *p* < 0.001, Fig. 3C, D). Collectively, these results suggest that 800 μM UA notably facilitates urate deposition, promotes LEC senescence, and contributes to cataract formation in rat lens organ cultures.

### MCC950 application alleviates UA-induced cellular senescence and cataract

**Fig. 4:**
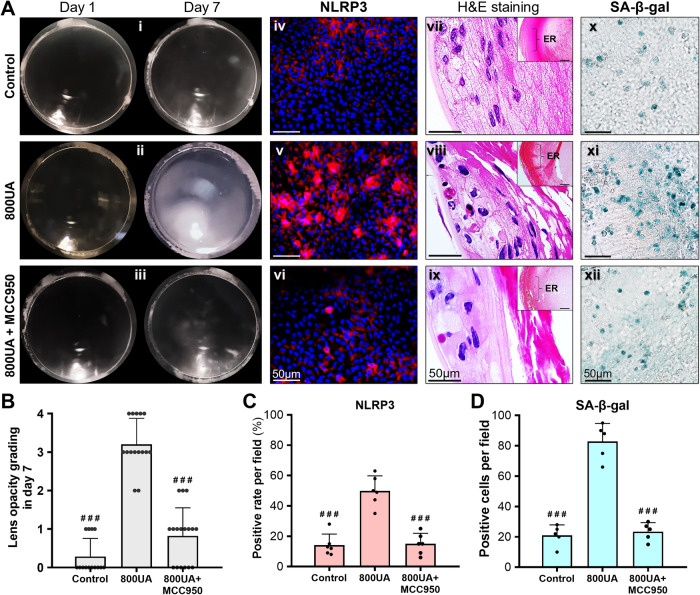
Blockade of the NLRP3 inflammasome reduced UA-driven LEC senescence and lens opacification on day 7 of organ culturing. **A** Co-treatment with 400 μM MCC950 postponed ex vivo cataract in rat lenses treated with 800 μM UA (i-iii). The histological sections were stained with NLRP3 (iv–vi), H&E (vii–ix), and SA-β-gal (x–xii). Scale bars are shown in the figure. ER equatorial region, LEC lens epithelial cell, 800UA 800 μM uric acid treatment. **B** Quantification of lens opacities on day 7 showed a significant reduction in the 800UA + MCC950 group compared with those in the 800UA group. Mean ± SD, *n* = 15. **C**, **D** Following NLRP3 decline after MCC950 application, SA-β-gal labeling markedly decreased. There were statistical differences in the NLRP3- and SA-β-gal-positive cells in the 800UA and 800UA + MCC950 groups. Mean ± SD, *n* = 5. ^#^*p* < 0.05, ^##^*p* < 0.01, ^###^*p* < 0.001 compared with the 800UA group.

Given that LEC senescence and lens opacification were caused by UA-activated NLRP3/caspase-1/IL-1β signaling, we investigated whether MCC950, a selective NLRP3 antagonist, could reverse these phenomena. We found that co-treating rat lenses with 800 μM UA and 400 μM MCC950 significantly reduced these phenomena compared to the 800UA group (Fig. 4Ai–iii). The opacity score on day 7 in the 800UA + MCC950 group decreased to 0.94 ± 0.83, similar to controls (0.29 ± 0.47, *p* = 0.0635), but the score was markedly lower than in the 800UA group (3.20 ± 0.68, *p* < 0.0001, Fig. 4B). Immunofluorescence (Fig. 4Aiv–vi) showed that MCC950 significantly inhibited UA-induced NRLP3 expression (*p* < 0.0001), reducing it to untreated levels (*p* = 0.9829, Fig. 4C). H&E staining revealed that MCC950 application restored normal LEC morphology, while elongated nuclei and irregular lens fiber cells were observed in the 800UA group (Fig. 4Avii–ix). Immunohistochemistry (Fig. 4Ax–xii) revealed that the ratio of SA-β-gal-labeled cells in the 800UA + MCC950 group was significantly lower than in the 800UA group (23.40 ± 5.94 vs. 82.80 ± 11.95%, *p* < 0.0001) and close to control levels (21.00 ± 6.87%, *p* = 0.9004, Fig. 4D).

**Fig. 5:**
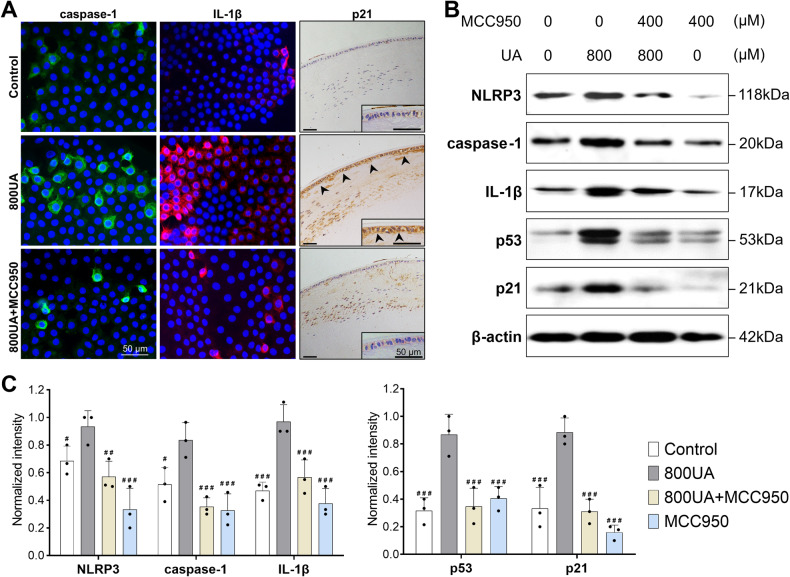
MCC950 application delayed senescence-related activation of the p53-p21 pathway by inhibiting NLRP3/caspase-1/IL-1β signaling. **A** Immunostaining microscopy revealed the activities of downstream effectors, visualized in green (caspase-1), red fluorescence (IL-1β), and brown puncta (p21; shown by the arrowheads) (scale bars = 50 μm). **B**, **C** Expression levels of inflammasome and senescence markers on day 7 were investigated with a Western blot followed by corresponding densitometry analysis. Inhibition of the NLRP3 inflammasome with MCC950 suppressed the downstream expression of cleaved caspase-1 and mature IL-1β, resulting in a significant reduction of p53 and p21 expression. 800UA, 800 μM uric acid treatment. Mean ± SD, *n* = 3. ^#^*p* < 0.05, ^##^*p* < 0.01, ^###^*p* < 0.001 compared with the 800UA group.

To explore MCC950’s impact on downstream signaling, we assessed cleaved caspase-1 and mature IL-1β with immunofluorescence, and found that LECs in the 800UA group exhibited significantly stronger cytosolic fluorescence for caspase-1 and IL-1β than those in untreated controls, while MCC950 co-treatment markedly reduced them, to control levels (Fig. 5A). A similar reduction was observed in histological staining images for the senescence marker p21 (shown by the arrowheads in Fig. 5A). Western blot analysis also revealed a significant increase in NLRP3, caspase-1, and IL-1β proteins levels in the 800UA group (all *p* < 0.01), with MCC950 reversing this expression and restoring it to untreated levels (Fig. 5B, C). Following this inhibition, MCC950 further reduced the downstream expression of p53 and p21, which were highly expressed in the 800UA group (all *p* < 0.001, Fig. 5B, C). Overall, these findings highlight that blocking the NLRP3 inflammasome in LECs can counteract UA-induced cataract progression by reducing key signaling mediators and senescent biomarkers.

### In vivo assessment of NLRP3 inhibition in UA-induced cataractogenesis

**Fig. 6:**
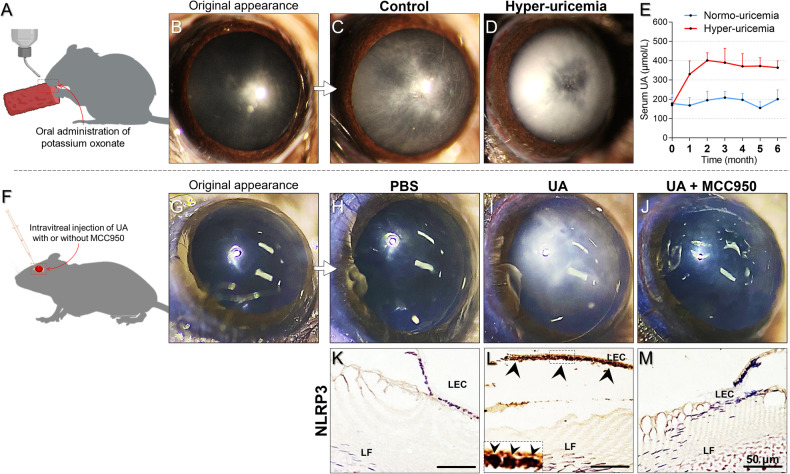
In vivo modeling of UA-induced cataractogenesis and the application of MCC950. **A**–**D** A mouse model of hyper-uricemia was established via continuous oral administration of potassium oxonate and maintained until lens opacification (*n* = 5 per group). **E** Blood samples were collected monthly from the tail vein to monitor serum UA levels. **F**–**J** Another model of UA-induced cataract was based on the direct intravitreal injection of 5 mM UA in mice, with some mice receiving a co-injection of 3 mM MCC950. Lens conditions were monitored daily until cataract phenotypes developed (*n* = 4 per group). **K**–M Corresponding lens sections from each group were stained for NLRP3 protein (shown by arrowheads) (scale bars = 50 μm). LEC lens epithelial cell, LF lens fiber, UA uric acid.

To validate our ex vivo findings, we developed an in vivo hyper-uricemia model using potassium oxonate (Fig. 6A), resulting in consistently sustained elevated serum UA levels (range from 330.47 to 401.27 μmol/L, Fig. 6E). Notably, after six months, a severe mixed cataract phenotype developed, in contrast to the mild cortical opacities observed in control lenses (Fig. 6B–D). Moreover, mice in the hyper-uricemia group exhibited emaciation, listlessness, and high mortality, which were not seen in controls. Due to these concerns, we explored an alternative model using intravitreal UA injection (Fig. 6F). Mice receiving 2 mM UA developed marked cortical cataracts within 21 days, but 2 mM MCC950 co-injection reversed the effect, and the mice retained clear lenses (Fig. 6G–J). Additionally, positive staining for NLRP3 in the lens epithelial tissues (shown by the arrowheads) was detected in the UA group but was absent in the PBS and UA + MCC950 groups (Fig. 6K–M). These results confirmed that excessive UA promotes cataract development and that NLRP3 inhibition can counteract this pathology, thus supporting our ex vivo findings.

## Discussion

**Fig. 7:**
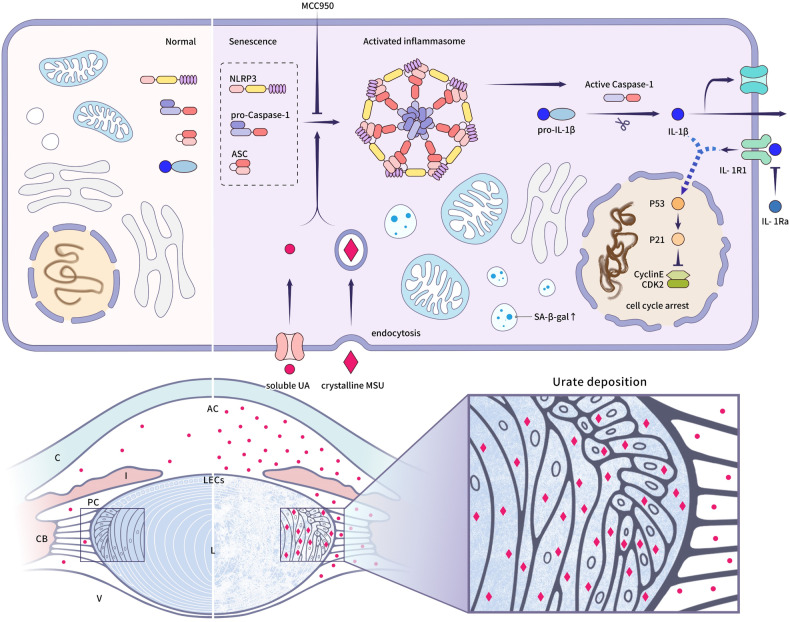
Schematic illustrating how excessive UA or urate deposition promotes LEC senescence and lens opacification via activation of the NLRP3 inflammasome. UA elevation in the aqueous humor causes urate deposition in the lenticular epithelium and cortex. Concurrently, soluble UA or crystalline MSU act on the lens epithelial cells, resulting in NLRP3 inflammasome activation. The inflammasome cleaves pro-caspase-1 to active caspase-1, which provokes an inflammatory response, represented by IL-1β maturation and release. Persistent inflammation leads to LEC senescence caused by p53-p21 upregulation and changes in the organelles. Consequently, sustained cellular senescence precipitates aging and opacification of the lens. L lens, C cornea, I iris, V vitreous body, CB ciliary body, AC anterior chamber, PC posterior chamber, LEC lens epithelial cell, UA uric acid.

The present study is the first to our knowledge to reveal the underlying mechanism of UA-induced cataract; we used an analysis of anterior capsule samples from human lenses and performed corroborating experiments in rat lens organ cultures. We found that the administration of 800 μM UA activated the NLRP3 inflammasome, cleaved effector caspase-1, and released the inflammatory factor IL-1β, which caused a persistent inflammatory response. This eventually resulted in LEC senescence and lens opacification. Moreover, blockading NLRP3 with MCC950 significantly reduced these reactions, thereby preventing cataract formation. Taken together, these findings underscore the critical role of the NLRP3 inflammasome in the pathology of UA-induced cataract (Fig. 7).

In our previous study, we used in vitro methods to illustrate the relationship between aqueous humor UA levels and cataract incidence, as evidenced by urate deposition and cellular senescence, in LECs treated with 200 μM UA [11]. Unexpectedly, in an ex vivo organ culture, treatment with 200 μM UA resulted in only limited rat lens clouding, indicating low repeatability at this concentration (Supplementary Fig. S2). Thus, we increased the UA dosage; we then observed stable lens opacities at 800 μM (Fig. 2). Given that soluble UA is applied at a dose of 15 to 50 mg/dL in some models (the limit of UA solubility in blood is 6.8 mg/dL) [15, 19, 20], it was not surprising that we had to use a higher concentration in ex vivo conditions within a short period. Additionally, the UA crystallization rate is influenced by environmental factors, such as temperature, pH, ions, serum factors, and growth factors [21–23], which are all absent in ex vivo conditions. It is challenging to achieve crystallization with low UA doses in lens cultures (Supplementary Fig. S3).

Urate deposition (also called MSU crystal precipitation), and not soluble UA, has long been regarded as the dominant trigger for inflammatory and immune responses, [13]. This might explain why only 10% of individuals with hyper-uricemia experience gout attacks [24]. MSU crystals activate a specific inflammatory cascade that produces pro-inflammatory cytokines and modulates the adaptive immune system via innate immune cells [25] while also promoting dendritic cell maturation by upregulating the co-stimulatory molecules CD80 and CD86 [26]. Zhou et al. reported that in hyper-uricemic mouse kidneys, urate accumulation induced macrophage infiltration and increased expression of pro-inflammatory cytokines, such as NF-κB, TNF-α, and MCP-1 [27]. Moreover, IL-1β cleavage is also a classical manifestation of MSU-driven inflammation, which depends on the mediation of the NLRP3 inflammasome [16]. We analyzed UA-related RNA-seq data from the GEO dataset (GSE65931) and found that UA is closely associated with cytokine–cytokine receptor interaction and the NOD-like receptor signaling pathway, of which the NLRP3/caspase-1/IL-1β axis is a crucial component (Supplementary Figs. S4,S5). Our ex vivo data also confirmed that urate deposition in cultured lenses stimulated IL-1β production, and that NLRP3 inflammasome inhibition with MCC950 reversed this effect (Fig. 5).

As noted earlier, we applied soluble UA, not MSU crystals, to cultured lenses and observed urate deposition and lens opacities. Interestingly, the simultaneous presence of soluble urate and MSU crystals complicates the identification of the dominant factor driving NLRP3-mediated inflammation in cataract formation. Indeed, there is still controversy about whether soluble urate also activates NLRP3-dependent IL-1β production. Kim et al. reported that extracellular soluble UA aggravated diabetic kidney injury by activating the NLRP3 inflammasome in macrophages [19]. Xiao et al. found that soluble MSU, but not MSU crystals, triggered NLRP3 upregulation, IL-1β processing, and CD40 overexpression in human mesangial cells via toll-like receptor 4 [28]. Some studies suggested that soluble UA did not independently induce IL-1β production [20, 29], instead amplifying IL-1β inflammatory effects via IL-1Ra inhibition or by acting together with other reagents, such as lipopolysaccharides [20]. Therefore, further exploration of this issue is necessary.

In our study, NLRP3/caspase-1/IL-1β signaling in LECs was upregulated within 24 h after the application of 800 μM UA (Fig. 2), indicating a sustained inflammatory challenge prior to lens opacification. This inflammation is known to generate excessive cellular proliferation or epithelial-mesenchymal transition in the early phase, followed by cell senescence, apoptosis, or pyroptosis in later phases [30–33]. Our previous work showed that the number of senescent LECs in UA-treated lenses exceeded that in cells undergoing apoptosis, indicating that cell senescence is a major pathway in UA-induced cataract [11]. Bogeska et al. reported that inflammation in early life accelerated cellular aging and molecular damage to hematopoietic stem cells, resulting in the failure of cellular function recovery [32]. This persistent increase in low-grade inflammation during aging, also known as “inflammageing,” is a hallmark of cell senescence [34]. Supporting this, we also identified SA-β-gal-positive LECs in anterior capsule specimens from hyper-uricemia patients and UA-cultured lenses (Fig. 1). Nonetheless, other mechanisms may also be involved; we thus conducted bulk RNA sequencing of lens capsular samples from organ cultures. This analysis identified key pathways, including the cell cycle, chromosome segregation, PI3K-Akt signaling, cytokine–cytokine receptor interaction, and leukocyte trans-endothelial migration (Supplementary Fig. S6), which is consistent with our experimental findings and the concept of “inflammageing.” The data also indicated other mechanisms involved in UA-induced cataract, such as cell migration and the epithelial-mesenchymal transition. In future studies, we are planning to sequence clinical samples from hyper-uricemic patients to better understand UA-induced cataractogenesis. Because of the challenges in tissue collection during phacoemulsification, we need to explore more viable alternatives to obtain sufficient samples of lens capsules for high-throughput analysis.

Cell senescence, induced by various stresses, is characterized by irreversible cell cycle arrest, along with secretory features, a dysregulated metabolism, and macromolecular damage [35], which are crucial to cataract pathology. For example, in the aging lens, increasing damage from PLAAT phospholipases causes lens deterioration stemming from the inhibition of organelle degeneration [36]. Senescent cells are identified by various phenotypes and hallmarks, like SA-β-gal, p53, p16^INK4a^, p21^CIP1^, lamin B1, or retinoblastoma (Rb), but presently SA-β-gal is the most commonly used marker, directly indicating a lysosomal stress response [30, 37]. Additionally, DNA damage–induced p53 activation inhibits cyclin-dependent kinases by initiating p21, leading to cell senescence [38]. Moreover, the p53-p21 axis is a well-accepted effector pathway in organism aging that retains the active form of Rb protein and leads to cell-cycle arrest [39]. Consistent with this, p53 knockdown can partly postpone aging phenotypes induced by 1,25(OH)2D3 deficiency [40], and silencing p53 almost completely abolishes plasminogen activator inhibitor 1 (PAI-1)–induced p21 expression and cell senescence [41]. Our findings also corroborate findings on UA-driven senescence involving SA-β-gal-positive staining and p53-p21 signaling in LECs, which is reduced upon NLRP3 inflammasome disruption by MCC950.

In our in vivo study, we explored UA-induced cataract models using two approaches: inducing hyper-uricemia in mice and administering intraocular injections of UA. The hyper-uricemia model, while effective, required us to wait over 6 months for the animals to develop cataract phenotypes (Fig. 6A–E), and it also led to health risks and high mortality. Therefore, we could not use this model to investigate other interventions, such as NLRP3 knock-out or MCC950 eye drops. Instead, we used direct intraocular UA injections, which rapidly induced cataract phenotypes (within 21 days); we found that MCC950 co-injection reduced the incidence of these cataract phenotypes (Fig. 6F–J). To our knowledge, we are the first to present findings from an in vivo model of UA-induced cataract; moreover, our findings support the results of our ex vivo experiments. Nevertheless, we note that it is still necessary to enhance the model’s safety and repeatability in future studies.

In summary, our clinical discoveries suggest that we have developed an efficient lens culture model to elucidate the mechanisms underlying UA-induced cataract. As illustrated in Fig. 7, excessive environmental UA activates the intracellular NLRP3 inflammasome, leading to persistent IL-1β maturation and release, ultimately resulting in LEC senescence and lens opacification. This study is the first to delineate the relationship between NLRP3/caspase-1/IL-1β signaling and UA-driven cataract. Our findings suggest that selectively targeting the NLRP3 inflammasome could be a promising therapeutic approach to delay cataract progression.

## Materials and methods

### Patient enrollment and specimen collection

Patients with age-related cataract and hyper-uricemia (*n* = 7) or normo-uricemia (*n* = 7) were selected as representative samples. This study was approved by the Institutional Human Research Ethics Committee of Guangdong Provincial People’s Hospital (petition No. KY-Z-2021-041-01) and written informed consent was obtained from the patients. Cataract severity was graded by ophthalmologists using the Lens Opacity Classification System III. Aqueous humor samples (100 μL) and blood samples were collected with a procedure described in a previous study [11]. Anterior capsule tissue was acquired during capsulorhexis and immediately fixed in 0.9% normal saline or 10% formalin for subsequent experiments.

### Animals

The Guangdong Medical Laboratory Animal Center provided 10-week-old C57BL/6J male mice and 16-week-old Sprague-Dawley (SD) male rats. All animals were fed ad libitum and maintained under a 12-h light/dark cycle for 1 week. Related experiments conformed to the Association for Research in Vision and Ophthalmology (ARVO) statement and were approved by the Animal Ethics Committee of Guangdong Provincial People’s Hospital (petition No. KY-Z-2021-069-01).

### Organ culturing of rat lenses and drug administration

Sixty male SD rats underwent lens isolation and organ culturing as previously described [42, 43]. Briefly, the rats were humanely killed with a carbon dioxide (CO_2_) euthanasia apparatus. Their eyes were dissected posteriorly, and lenses were extracted and cultured in M-199 medium (M5017, Sigma-Aldrich, St. Louis, USA) containing 5% fetal bovine serum (FBS, Gibco, Grand Island USA) and antibiotics (100 U/mL penicillin, 100 μg/mL streptomycin, 0.25 μg/mL amphotericin B). The cultured lenses were treated with increasing doses (200 to 1000 μM) of soluble UA (U2625, Sigma-Aldrich) to determine the effective dose for inducing cataract formation. Subsequent experiments used the established model to test the effects of 400 μM MCC950 (CSN18163, CSNpharm, Chicago, USA), a selective NLRP3 antagonist [44]. The lenses were cultured at 37 °C in 5% CO_2_ for a week with daily lens opacity assessments (Supplementary Table S1 and Fig. S1). “Clear lens survival” was defined as an opacity grade ≤1 under microscopy. Additionally, lenses were collected at 12, 24, or 48 h to measure NLRP3/caspase-1/IL-1β signaling. Eventually, all lenses underwent Western blot and histological analysis.

### Immunofluorescence and quantification

Capsular epithelial samples from human cataract surgery or rat lens organ cultures were immediately fixed in 4% vol/vol paraformaldehyde, permeabilized with 0.5% vol/vol Triton X-100, and washed with PBS. The samples were then blocked with 5 wt/vol goat serum to neutralize nonspecific binding sites. Following flat-mounting on slides, as described previously [45], the explants were incubated with primary antibodies against NLRP3 (1:50, NBP2-12446, Novus Biologicals, Centennial, USA), caspase-1 (1:50, NBP1-45433, Novus Biologicals), or IL-1β (1:50, ab283818, Abcam, Cambridge, UK) overnight at 4 °C. After PBS rinsing, they were conjugated with Alexa-Fluor 488 or 594 secondary antibodies (both 1:200, ab150077 and 150080, Abcam) for 1 h at room temperature. Finally, the explants were mounted with DAPI staining media (Santa Cruz, Dallas, USA), and imaged using a fluorescence microscope (Zeiss, Oberkochen, Germany). Positive cells were counted in three randomly selected fields per section by a blinded observer.

### SA-β-gal staining

SA-β-gal, a senescence biomarker, was stained with a senescent-cell histochemical staining kit (CS0030, Sigma-Aldrich) according to the manufacturer’s protocol. The lens anterior capsule tissue samples taken from cataract surgery patients or rat lens organ cultures were immediately fixed for 15 min at room temperature and then flat-mounted on slides. Subsequently, the explants were incubated in a staining solution at 37 °C overnight to reveal the localization of SA-β-gal. Positively stained senescent LECs appeared blue under a light microscope (Zeiss). The ratio of SA-β-gal-positive cells in each sample was counted in five random fields of view by a masked investigator.

### Transmission electron microscopy

Rat lenses treated with or without 800 μM of UA for 7 days were fixed in 2.5% wt/vol glutaraldehyde in 0.1-M phosphate buffer (pH 7.5) overnight at 4 °C, and the lenses were then postfixed in 1% wt/vol osmium tetroxide (OsO_4_, Sigma-Aldrich) for 2 h. Then, after being dehydrated in a graded concentration of ethanol, the lenses were embedded in epoxy resin and cut into thin sections (50 nm). The sections were stained with uranyl acetate and lead citrate before images were captured with transmission electron microscopy (JEM-2100F; JEOL, Tokyo, Japan).

### Histological evaluation

After organ culturing, selected lenses were sectioned for hematoxylin and eosin (H&E, C0105S, Beyotime) staining and urate deposition staining with Gomori methenamine silver (GMS, BB-44715, Bestbio, Shanghai, China), as we previously reported [11, 46]. The lenses were fixed in 4% paraformaldehyde, dehydrated, embedded in paraffin, and sectioned coronally at 5 μm thickness. Each section included the anterior, equator, and posterior positions. After deparaffinization and rehydration, some sections were stained with H&E for morphological observation, and others were conjugated with GMS for uric acid detection.

### Western blot analysis

Capsular epithelial samples from cultured lenses were isolated, and six samples per group were collected together in one tube to ensure an adequate protein level. The samples were lysed in a RIPA extraction buffer containing protease inhibitors (BB-3101, Bestbio) and quantified using a BCA Protein Assay Kit (#23225, Thermo Fisher Scientific, Waltham, USA). The protein samples were subjected to electrophoresis and electroblotted onto membranes as previously described [47]. Membranes were then blocked with 5% milk and incubated overnight with primary antibodies targeting NLRP3 (1:500, NBP2-12446, Novus Biologicals), caspase-1 (1:200, NBP1-45433, Novus Biologicals), IL-1β (1:1000, ab283818, Abcam), p21 (1:1000, ab188224, Abcam), p53 (1:200, ab26, Abcam), and GAPDH (1:1000, #3683, Cell Signaling Technology, Danvers, USA), followed by a secondary HRP-conjugated antibody. Protein bands were visualized using enhanced chemiluminescence (BC-WB-004, Biochannel, Nanjing, China) and analyzed with ImageJ 2.0 software, with band densities normalized to β-actin. Full Western blot images are attached as a supplemental file.

### In vivo modeling of UA-induced cataract

Ten C57BL/6J mice were randomly allocated into a control group and a hyper-uricemia group (*n* = 5 per group). Mice in the hyper-uricemia group received continuous oral administration of potassium oxonate, a uricase inhibitor, until cataract was observed. The control mice were fed a standard diet. Lens evaluation and blood sampling were conducted monthly, with blood specimens sent to Guangdong Provincial People’s Hospital for serum UA examination.

In a separate experiment, 12 mice were randomized to three groups: PBS, UA, and UA + MCC950 (*n* = 4 per group). The groups received an intravitreal injection of either 2 μL of PBS, 2 mM UA, or 2 mM UA plus 2 mM MCC950, respectively. Injections were administered once every 7 days. Any mice exhibiting lens injuries or intraocular hemorrhages during the operation were excluded. Lens conditions were monitored daily until the onset of cataract, after which the lenses were collected for NLRP3 immunostaining.

### Statistical analysis

Each experiment was performed at least three times, with data statistically analyzed using SPSS (version 20.0, Chicago, USA). Student’s *t*-test or the Mann–Whitney *U*-test were used to determine statistical differences between pairs of experimental groups, while a one-way analysis of variance (ANOVA) with a post hoc Tukey’s test was used for comparisons of multiple groups. Results are presented as mean ± standard deviation (SD), with a *p* value less than 0.05 considered statistically significant.

### Supplementary information

Supplementary Tables & Figures

Uncropped WB

## Data Availability

The datasets generated during and/or analyzed during the current study are available from the corresponding author on reasonable request.
