# Supplementary material for: Uric acid–driven NLRP3 inflammasome activation triggers lens epithelial cell senescence and cataract formation
Source: Cell Death Discov. 2024 Mar 9;10:126. doi: 10.1038/s41420-024-01900-z (PMC10925029; doi:10.1038/s41420-024-01900-z)
Supplement: Supplementary file 1 — Supplementary Tables & Figures [file 41420_2024_1900_MOESM1_ESM.docx]

**Uric acid-driven NLRP3 inflammasome activation triggers lens epithelial cell senescence and cataract formation**

**Hong Liang Lin ^#^, Sheng Wang^#^, Kota Sato, Yu Qiao Zhang, Bei Ting He, Toru Nakazawa, Yong Jie Qin*, and Hong Yang Zhang***

*** Correspondence:**Hong Yang Zhang (Email: hy3005716@163.com)
Yong Jie Qin (Email: qyjie@link.cuhk.edu.hk)

# Supplementary Figures and Tables

## Supplementary Tables

| **Supplementary Table S1.** Lens opacities grading in organ culture | | | |  |
| --- | --- | --- | --- | --- |
| Grade 0 |  | Clear lens | | |
| Grade 1 |  | Mild change (Slight haze) | | |
| Grade 2 |  | Partial opacity (Line just visible) | | |
| Grade 3 |  | Dominating opacity (Line partially visible) | | |
| Grade 4 |  | Completely opaque (Line invisible) | | |
| The grading criterion were referred to a previous literature.  (Hernebring M., Exp Eye Res, 2020 and Bree M., Exp Eye Res. 2018) | | |  |  |

## Supplementary Figures


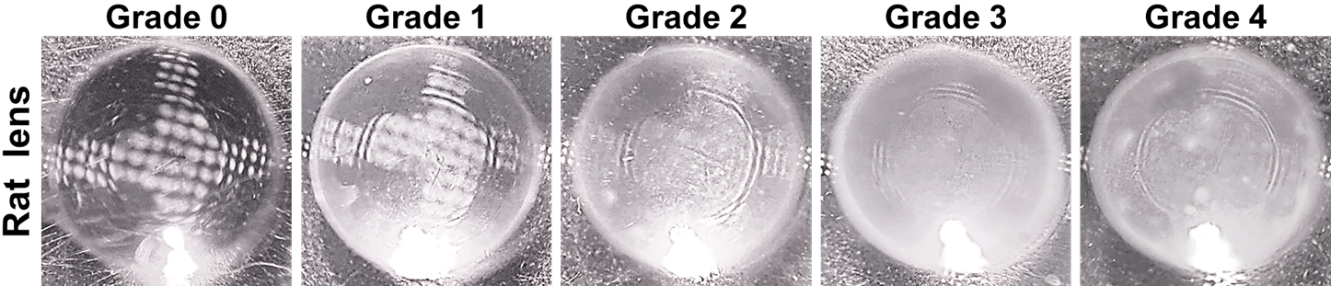


**Supplementary Figure S1.** Photograph of a rat lens showing lens opacity grading. Detailed criteria for lens grading are described in Supplementary Table S1.


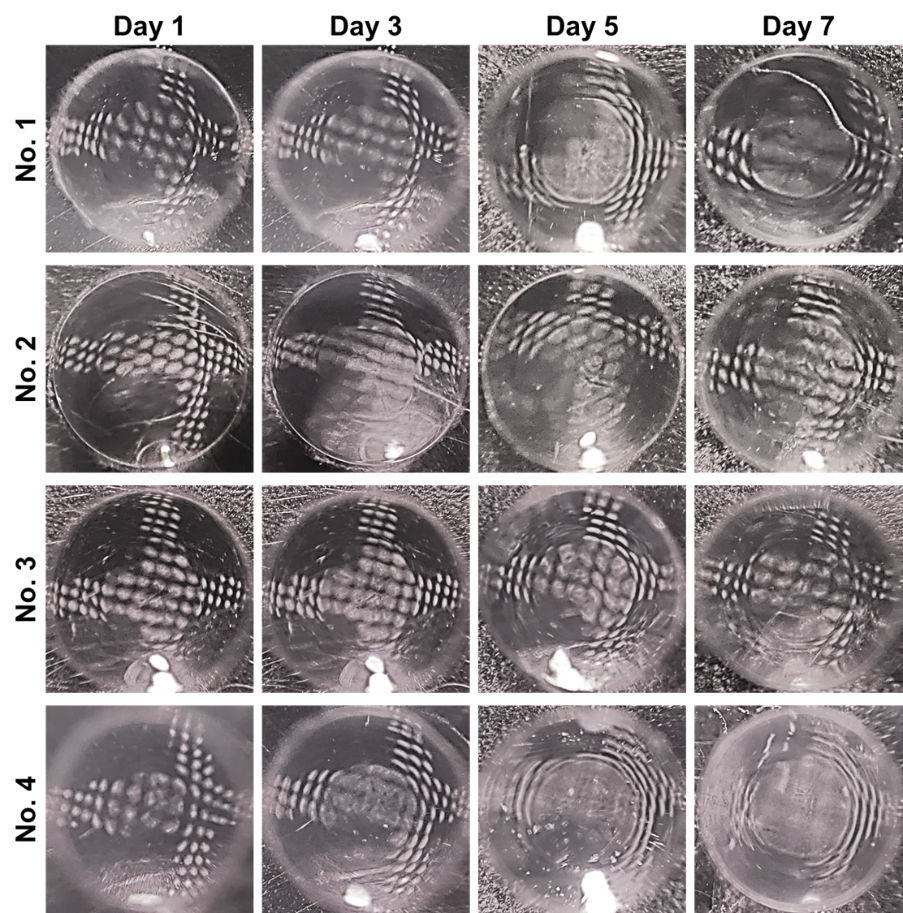


**Supplementary Figure S2.** Photograph of four groups of rat lenses incubated with 200 μM of UA. Visible opacity was rarely observed after 7 days of incubation.


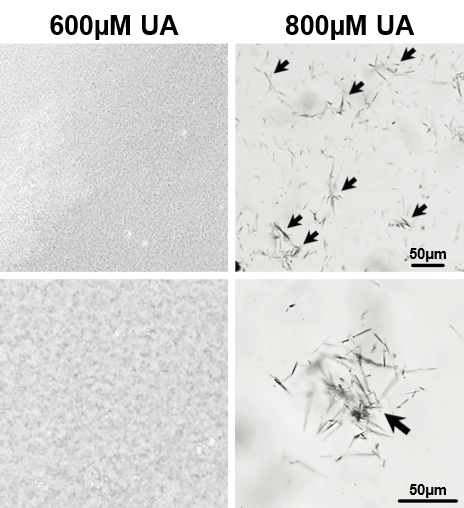


**Supplementary Figure S3.** Microscope image showing the crystallization of monosodium urate (MSU) after 600-μM and 800-μM UA treatment. Interestingly, visible MSU crystals were present in the 800-μM UA solution, but not the 600-μM UA solution (scale bars = 50 μm).


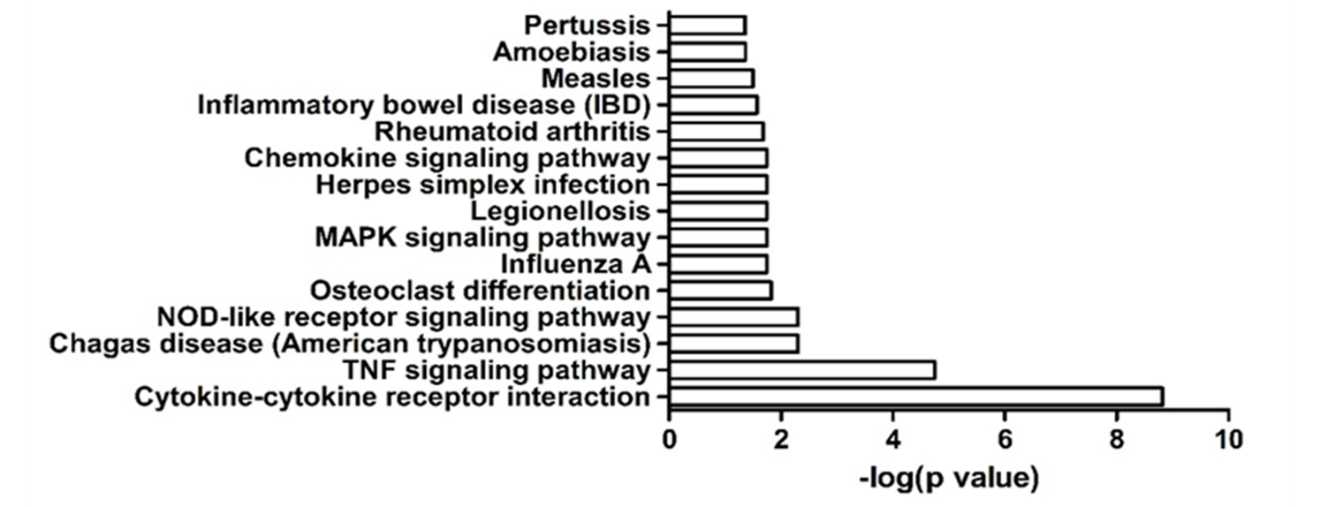


**Supplementary Figure S4.** UA-related RNA-seq data was retrieved from GEO dataset (GSE65931), and related gene ontology enrichment analysis demonstrated that the closest relationships of the UA-driven response, like cytokine–cytokine receptor interactions & NOD-like receptor signaling pathway.


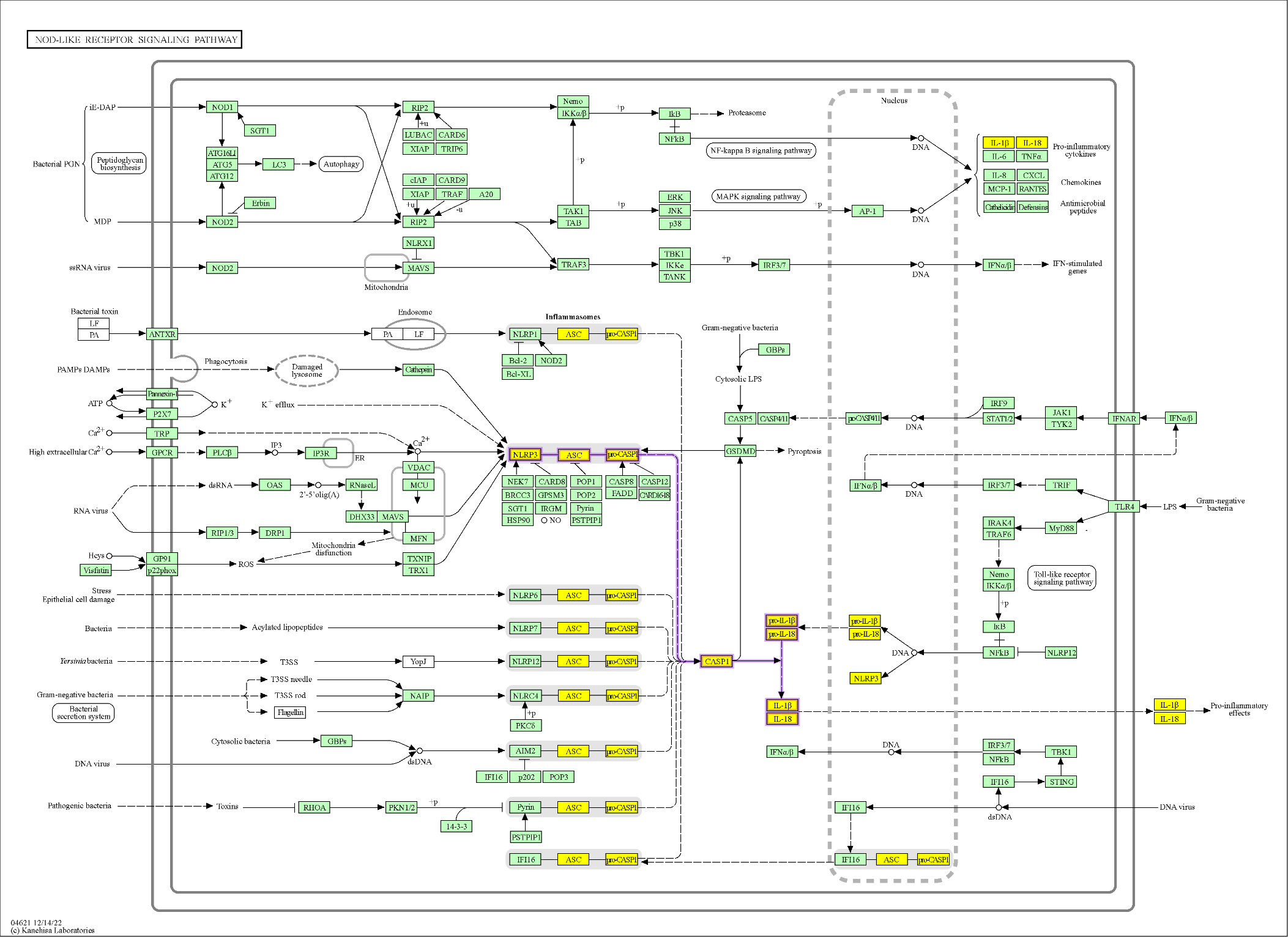


**Supplementary Figure S5.** Visualization of NOD-like receptor signaling pathway (hsa04621) was retrieved from KEGG pathway database. NLRP3/caspase-1/IL-1β signaling was marked with yellow background and purple line.


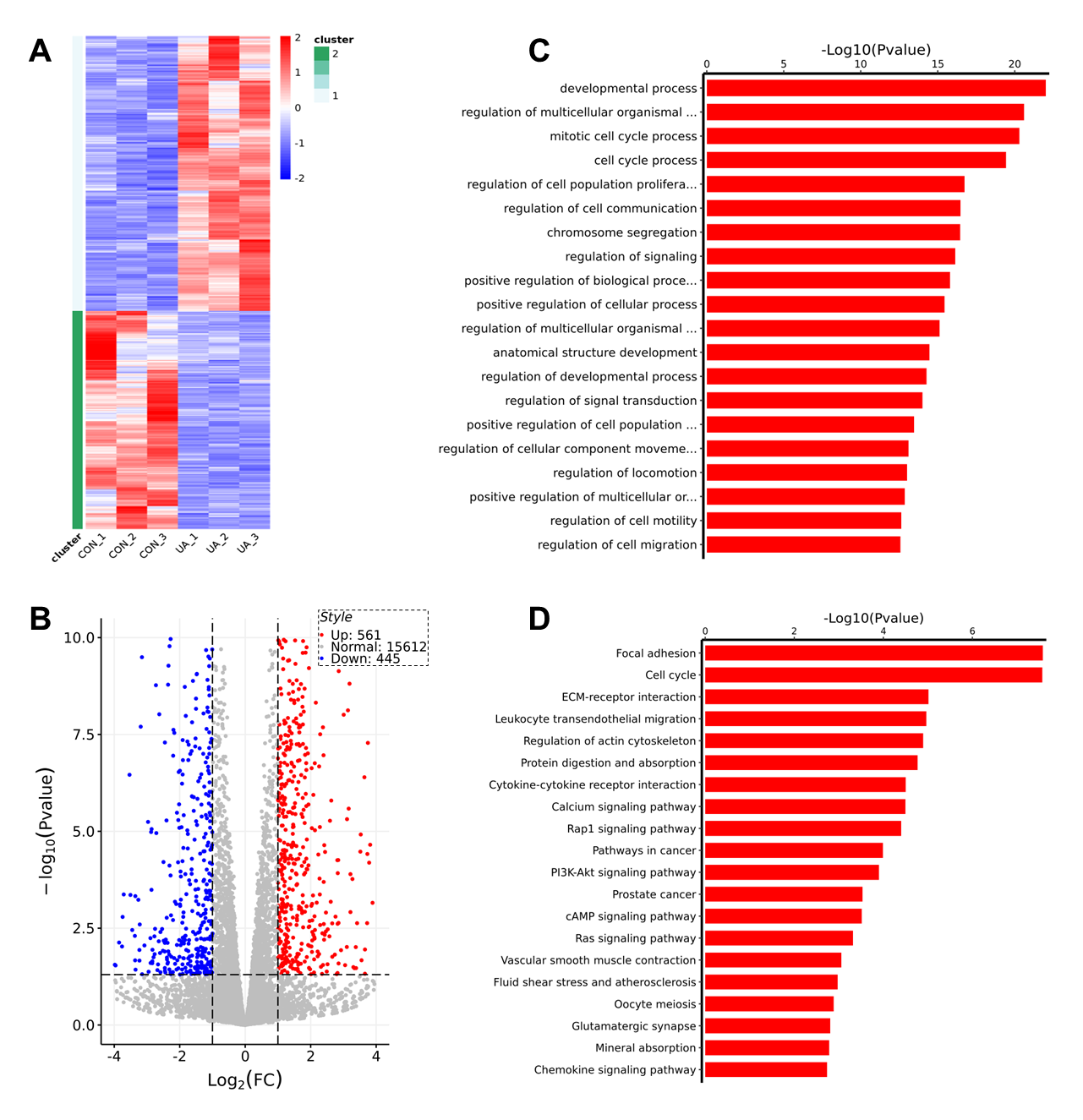


**Supplementary Figure S6.** Comparison of transcriptomes of untreated lens and UA-treated lens in ex vivo organ culture. RNA sequencing and data analysis were performed by Shanghai Jiayin Biotechnology Ltd. (A) Heatmap showing gene expression changes of LECs after UA induction (adjusted *p*<0.05). (B) Volcano plot visualizing the significantly altered, up- and down-regulated, genes in untreated LECs versus UA-treated LECs. *p*-values (−log_10_ *p*) plotted against fold changes (log_2_FC). (C) GO and (D) Pathway enrichment analysis in UA-induced LECs, showing the role of cell cycle alteration in UA-induced cataract.
