# Supplementary material for: Uric acid–driven NLRP3 inflammasome activation triggers lens epithelial cell senescence and cataract formation
Source: Cell Death Discov. 2024 Mar 9;10:126. doi: 10.1038/s41420-024-01900-z (PMC10925029; doi:10.1038/s41420-024-01900-z)

Fig. 2E

NLRP3 (118kDa)

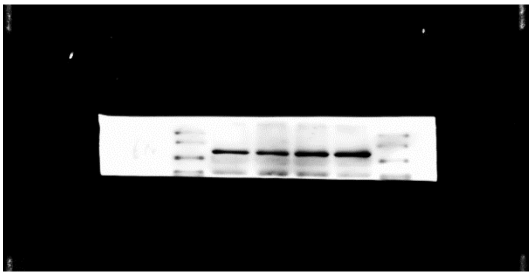

Caspase-1 (20kDa)

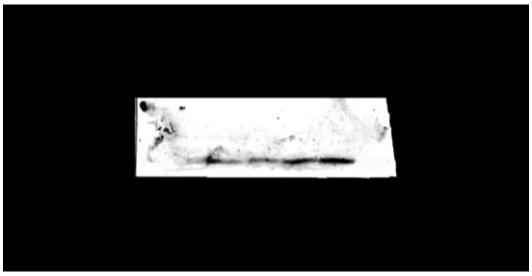

IL-1β (17kDa)

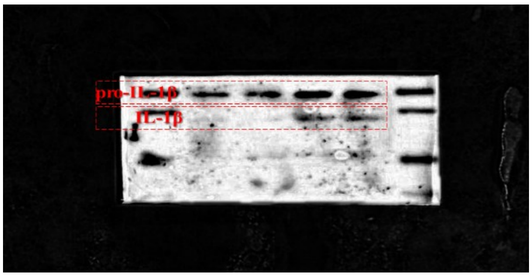

β-actin (42kDa)

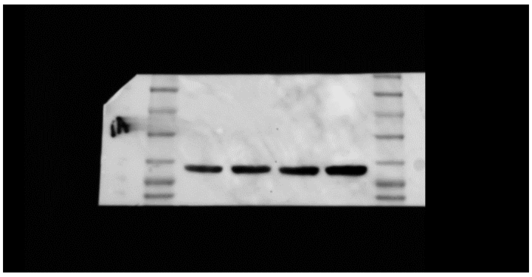

Fig. 2G

NLRP3 (118kDa)

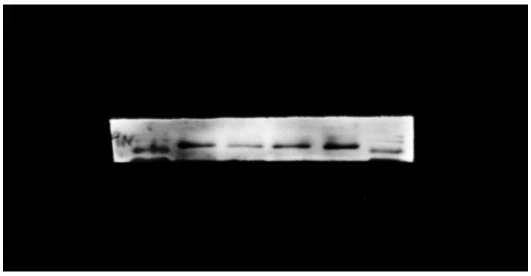

IL-1β (17kDa)

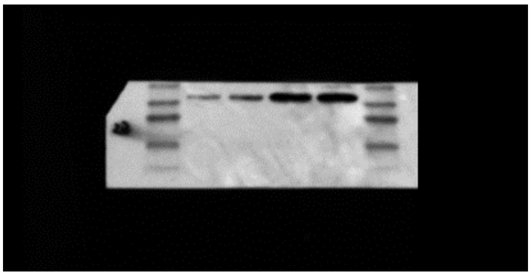

Caspase-1 (20kDa)

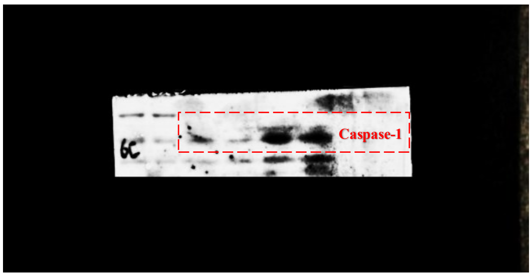

β-actin (42kDa)

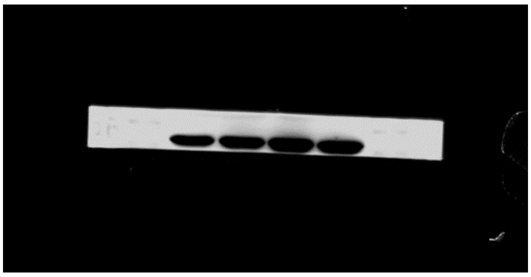

Fig. 3C

p53 (53kDa)

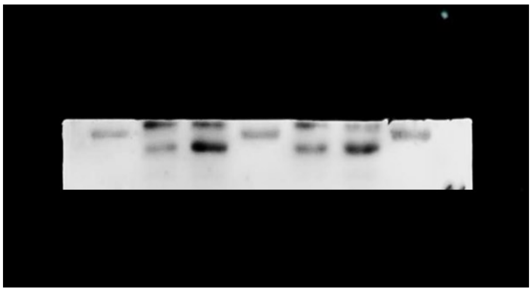

p21 (21kDa)

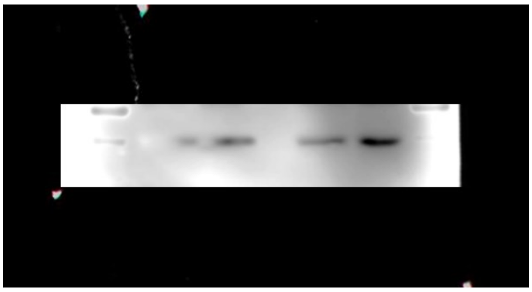

β-actin (42kDa)

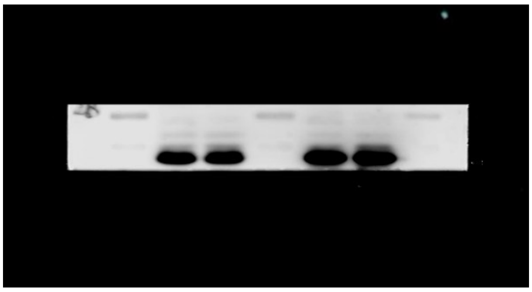

Fig. 5B

NLRP3 (118kDa)

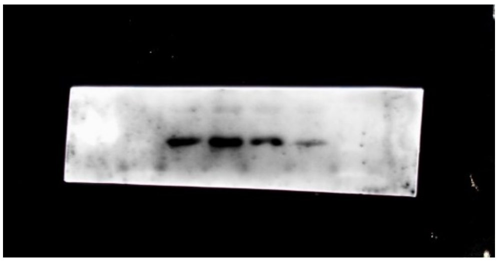

Caspase-1 (20kDa)

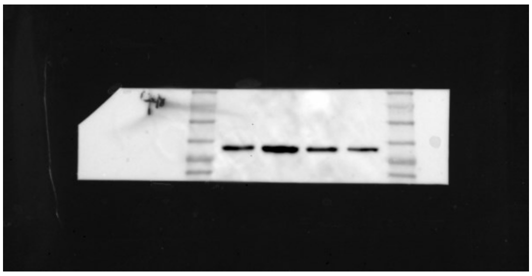

IL-1β (17kDa)

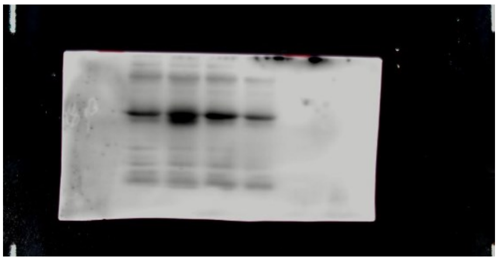

p53 (53kDa)

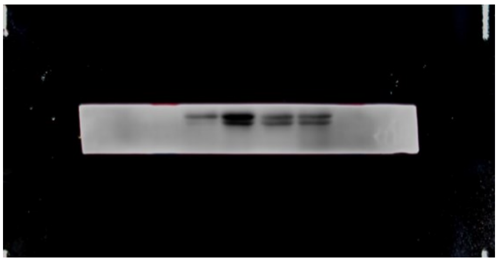

p21 (21kDa)

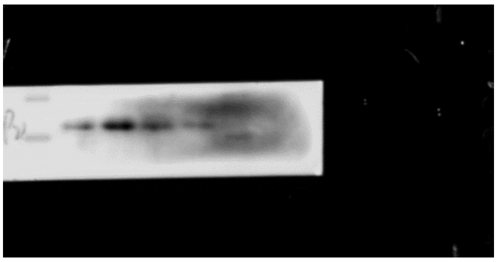

β-actin (42kDa)

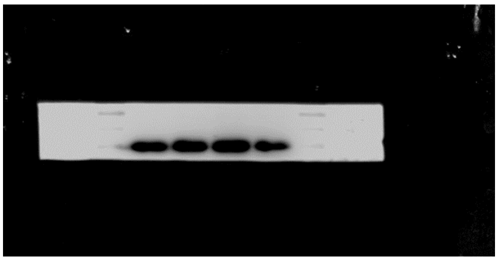

Supplement: Supplementary file 2 — Uncropped WB [file 41420_2024_1900_MOESM2_ESM.pdf]
